# Nutritional Regulators of Bcl-xL in the Brain

**DOI:** 10.3390/molecules23113019

**Published:** 2018-11-19

**Authors:** Han-A Park, Katheryn Broman, Allison Stumpf, Sara Kazyak, Elizabeth A. Jonas

**Affiliations:** 1 Department of Human Nutrition and Hospitality Management, College of Human Environmental Science, The University of Alabama, Tuscaloosa, AL 35487, USA; kabroman@crimson.ua.edu (K.B.); aastumpf@crimson.ua.edu (A.S.); srkazyak@crimson.ua.edu (S.K.); 2 Department of Biological Sciences, College of Arts and Sciences, The University of Alabama, Tuscaloosa, AL 35487, USA; 3 Department of Internal Medicine, Section of Endocrinology, Yale University, New Haven, CT 06520, USA; elizabeth.jonas@yale.edu

**Keywords:** Bcl-xL, mitochondria, neuroprotection, nutrients

## Abstract

B-cell lymphoma-extra large (Bcl-xL) is an anti-apoptotic Bcl-2 protein found in the mitochondrial membrane. Bcl-xL is reported to support normal brain development and protects neurons against toxic stimulation during pathological process via its roles in regulation of mitochondrial functions. Despite promising evidence showing neuroprotective properties of Bcl-xL, commonly applied molecular approaches such as genetic manipulation may not be readily applicable for human subjects. Therefore, findings at the bench may be slow to be translated into treatments for disease. Currently, there is no FDA approved application that specifically targets Bcl-xL and treats brain-associated pathology in humans. In this review, we will discuss naturally occurring nutrients that may exhibit regulatory effects on Bcl-xL expression or activity, thus potentially providing affordable, readily-applicable, easy, and safe strategies to protect the brain.

## 1. Introduction

The Bcl-2 family of proteins are best known for their role in apoptosis by means of mitochondrial membrane permeabilization and subsequent caspase regulation. Bcl-2 proteins are structurally similar in that they all share one or more of four Bcl-2 homology (BH) domains: BH2, BH1, BH3, and/or BH4. Generally, the Bcl-2 proteins are divided into two groups, anti-apoptotic and pro-apoptotic. Anti-apoptotic proteins such as Bcl-2 and Bcl-xL contain all four homologues. Among four BH domains, the BH4 domain has been shown to be essential for the anti-apoptotic properties of Bcl-2 and Bcl-xL by its ability to inactivate pro-apoptotic effector proteins such as Bax [1,2]. Mutation on the BH4 domain reduces stability of Bcl-2 protein and interferes occupation of Bax by Bcl-2 [3]. In addition to its ability to heterodimerize pro-apoptotic Bcl-2 proteins, the BH4 domain is reported to interact with the C-terminal of inositol 1,4,5-trisphosphate receptor (IP3R) inhibiting calcium-mediated apoptosis [4]. Pro-apoptotic proteins are further classified into two groups: multidomain and BH3-only. The multidomain pro-apoptotic proteins include Bax and Bak. Once activated by BH3-only proteins, Bax and Bak promote apoptosis by transitioning from inactive monomers to oligomers, leading to the permeabilization of the mitochondrial outer membrane. Additionally, Bax and Bak can be inhibited by the anti-apoptotic members of the Bcl-2 family [5]. BH3-only proteins are unique in their pro-apoptotic mechanisms; the activator such as Bid promotes oligomerization of Bax and Bak [6,7,8], whereas the sensitizers such as PUMA and Bad sequestrate anti-apoptotic Bcl-2 proteins favoring apoptotic signaling [9].

B-cell lymphoma-extra large (Bcl-xL) is a mitochondrially localized, anti-apoptotic member of the Bcl-2 protein family. Bcl-xL has been shown to bind with pro-apoptotic Bcl-2 proteins such as Bax, Bak and Bad, thereby preventing apoptotic signaling that leads to the opening of pro-apoptotic mitochondrial ion channels [10,11,12,13]. By doing so, Bcl-xL supports cell survival by inhibiting intrinsic death pathways such as cytochrome c release and apoptosome assembly [14,15,16]. Although Bcl-xL is best known for its pro-survival role in cancer cells, additional functions of Bcl-xL in the central nervous system (CNS) have been recently reported. Bcl-xL is highly expressed in various regions of the brain including hippocampus, cortex, and hypothalamus [17,18,19], and it is abundantly found in both neurons and glia [18,20,21,22]. During prenatal development, various Bcl-2 proteins, such as Bcl-2, Bax, and Bcl-xL are expressed in the brain. However, protein levels of Bcl-xL remain high after birth through adulthood, thus Bcl-xL may be the critical player to protect the adult brain [23]. Bcl-xL enhances intracellular energy metabolism by supporting the activity of the F_1_F_o_ ATP synthase [24]. Energy conservation by actions of Bcl-xL may prevent neuronal injury caused by oxygen or glucose deficits during pathological processes of the brain. Bcl-xL is also reported to promote formation of synapses and recycling of synaptic vesicles, both of which are necessary for neuronal plasticity and recovery [25,26,27]. Bcl-xL is required for the extension and branching of neurites which is essential for both normal brain development and brain recovery after a neurotoxic event [21]. Due to its importance in brain functions, alteration of Bcl-xL activities or expression levels is highly associated with pathological processes in the brain (Table 1) [28,29].

**Figure 1:**
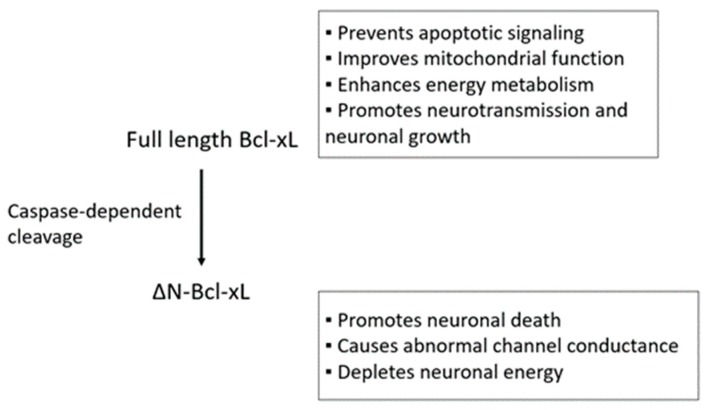
Conversion of Bcl-xL to ∆*N*-Bcl-xL. During neurotoxic stimulation (e.g., cerebral ischemia), full length Bcl-xL undergoes caspase-dependent *N*-terminus cleavage to pro-apoptotic ∆*N*-Bcl-xL.

In contrast to its protective role, Bcl-xL is also responsible for causing neuronal death during neurotoxic challenge. Bcl-xL undergoes caspase 3-dependent *N*-terminal cleavage forming ∆*N*-Bcl-xL (Figure 1) [65]. Loss of CA1 hippocampal neurons after cerebral ischemia is highly associated with the accumulation of ∆*N*-Bcl-xL in vivo [18,66,67]. Additionally, excitotoxicity, the major neuronal death signal during cerebral stroke caused by a surge of glutamate, leads to an abundance of ∆*N*-Bcl-xL in primary neurons in vitro [68]. Approaches that inactivate ∆*N*-Bcl-xL or prevent the accumulation of ∆*N*-Bcl-xL using cleavage resistant animal models, delivering pharmacological inhibitors, and applying ischemic preconditioning protect neurons by improving mitochondrial function against neurotoxic stimuli [18,66,67,68,69,70].

In addition to its role regulating the function of brain cells, Bcl-xL participates in intracellular signaling by interacting with various protein partners during physiological and pathological processes in cells [29]. Bcl-xL directly binds to the subunit of F_1_F_o_ ATP synthase [14,24]. Formation of a Bcl-xL-F_1_F_o_ ATP synthase complex prevents a proton leak through the c-subunit ring of F_1_F_o_ ATP synthase and increases intracellular ATP production [24,71]. Maintaining a functional population of neurons is energy-demanding: neurotransmission consumes a large amount of energy. Actin and tubulin polymerization during neuronal outgrowth require ATP and GTP, respectively. Therefore, Bcl-xL-dependent augmentation of neuronal energy status favors neurite outgrowth [21], synapse formation [26], and protection against ischemia-induced energy deficit [18,66]. 

Bcl-xL also directly binds to the IP3R located on the endoplasmic reticulum (ER) [72,73]. Opening of IP3R releases calcium from the ER to mitochondria regulating mitochondrial energy metabolism. Mitochondrial calcium influx controls activities of dehydrogenases such as pyruvate dehydrogenase [74,75,76], thus regulating mitochondrial NADH. Abundance of mitochondrial NADH increases availability of electrons supporting operation of the electron transport chain and synthesis of ATP. Studies have reported that calcium directly binds to F_1_F_o_ ATP synthase and regulates ATP production [77,78,79], and binding between calcium in large quantities and ATP synthase may also produce pathological mitochondrial membrane depolarization [80]. Together, the proximity between mitochondria and the ER regulates mitochondrial energy metabolism which is in turn regulated by Bcl-xL. This may further influence synaptic transmission and neuronal development. In addition, augmentation of ATP synthesis via formation of a Bcl-xL-F_1_F_o_ ATP synthase complex may influence IP3R activities. Since IP3R contains ATP binding sites and opening of the IP3R is regulated by different concentrations of ATP [81,82], Bcl-xL may also be responsible for controlling IP3R activation through enhanced production of ATP at sites closely apposed to the ER in the mitochondrial associated membrane (MAM). 

In addition to Bcl-xL and IP3R interaction, mitochondrial calcium influx may be regulated by Bcl-xL dependent voltage-dependent anion channel (VDAC) activities [83,84,85]. VDAC is expressed on the mitochondrial outer membrane, and it is reported to have multiple protein binding partners such as the adenine nucleotide translocator (ANT), hexokinase, and Bcl-2 family proteins. Formation of a multiprotein complex or oligomerization with other VDAC channels allows transport of ATP, ADP, NADH, metabolites (e.g., pyruvate, succinate), ions (e.g., Mg^2+^, and Ca^2+^), and cytochrome c [86,87]. Various groups have shown an interaction between Bcl-xL and VDAC [83,85,88,89], and formation of a Bcl-xL-VDAC complex can either enhance or inhibit activity of VDAC [90,91]. GST pulldown assay showed that VDAC 1 and 3 directly bind to both recombinant and endogenous Bcl-xL protein, and this interaction was associated with increases of mitochondrial calcium influx [83], whereas binding of the Bcl-xL BH4 domain with VDAC inhibited mitochondrial calcium uptake [85] via a reduction of channel conductance which may be associated with protection against apoptotic signaling [88].

Beclin-1, a critical player initiating autophagy [92,93,94], contains a BH3 domain. The crystal structure demonstrated a direct interaction between Beclin-1 and Bcl-xL, and occupation of Beclin-1 in the hydrophobic groove of Bcl-xL [95]. Dissociation of Bcl-xL and Beclin-1 induces autophagy in cancer cells [96], and mutation of the BH3 domain in Beclin-1 alters Bcl-xL-mediated autophagy [97] indicating regulatory roles of Bcl-xL in cell degradation. Interestingly, co-immunoprecipitation studies reveal that Bcl-xL also serves as a binding partner of PTEN-induced putative kinase 1 (PINK1), a mitochondrial serine/threonine kinase responsible for inducing mitochondrial autophagy (mitophagy) [98]. PINK1 phosphorylates ubiquitin, promotes recruitment of PARKIN and LC3, thus PINK1 facilitates formation of the autophagosome [98,99,100]. Alteration of PINK1 is highly associated with Parkinson’s disease [101,102] due to failure of mitochondrial quality control such as accumulation of impaired mitochondria. Although it is unknown how formation of a Bcl-xL-PINK1 complex directly influences mitophagy, PINK1 exhibits neuroprotective effects by phosphorylating serine 62 of Bcl-xL under apoptotic stimulation. PINK1-mediated phosphorylation of Bcl-xL inhibits *N*-terminal cleavage of Bcl-xL, thus preventing accumulation of neurotoxic Δ*N*-Bcl-xL [103].

Given these findings, Bcl-xL may be a necessary molecular player to maintain normal brain cell function, and it may be an important contributor to the delay and prevention of neurodegenerative processes [28,29]. Although it is necessary to pinpoint specific pathways of Bcl-xL-mediated neuroprotection using molecular approaches, currently reported strategies to manipulate Bcl-xL gene or protein may have limitations to translate into human subjects: Application of the pharmacological inhibitor (e.g., ABT-737) showed strong neuroprotective effects by blocking accumulation of Δ*N*-Bcl-xL but this drug does not cross the blood-brain barrier [18,68,104]. Overexpression of Bcl-xL improves neuronal survival [30,105] and Bcl-xL cleavage resistant animals protect the brain against ischemic injury [18], but genetic modification is still challenging to apply in human subjects. Currently, there are no FDA approved medications or treatments that specifically prevent Bcl-xL loss or promote Bcl-xL retention in human brain. In this review, we aim to introduce applicable strategies, nutritional intervention, that potentially regulate Bcl-xL protein, gene, or activity in the brain. Although limited information is available to explain a direct relationship between nutrients and Bcl-xL at the current time (Table 2), nutritional intervention may be an important extension in the field of Bcl-xL research due to its advantage in availability, low-toxicity, and cost-effectiveness. Here, we will discuss nutritional candidates that may participate in intracellular signaling pathways to delay degeneration of neurons or to promote recovery after neurotoxic insults via controlling expression of Bcl-xL.

## 2. Soy and Soy Isoflavones

Isoflavones are found predominantly in the legume family of flowering plants. In the human diet, soy isoflavones are often consumed in the form of soy or soy products, with the primary isoflavones being genistein, daidzein, and glycitein [128,129]. Soy isoflavones are called phytoestrogens due to chemical similarity with 17β-estradiol [130]. Studies have shown that soy isoflavones exert moderate levels of hormone-like effects or antagonize the interaction between estrogen and the estrogen receptor [131,132]. 

Soy isoflavones are reported to cross the blood-brain barrier [133]. Various research groups have reported improvement of the anti-apoptotic Bcl-2 protein profile in the brain after consumption of a soy diet or soy isoflavones in neurodegenerative models such as stroke, Alzheimer’s, and Parkinson’s disease [107,108,134,135,136]. Supplementation with soy isoflavones supports neuronal survival via enhancing Bcl-xL mRNA or protein expression in rodent brains [106,107,108,137]. Lovekamp-Swan et al. showed that rats supplemented with 600 µg/g soy isoflavones for 2 weeks prior to induction of middle carotid artery occlusion (MCAO), an experimental model to mimic focal ischemia, reduced MCAO-induced infarct size and protected brain cells from apoptotic death [107]. This group also showed that animals fed with a high soy diet had significantly enhanced mRNA and protein levels of Bcl-xL, without increasing Bcl-2 expression [107]. Interestingly, a high soy diet downregulated active caspase 3, the major contributor to the conversion of anti-apoptotic Bcl-xL to pro-apoptotic ∆*N*-Bcl-xL. Thus, oral supplementation of soy may prevent apoptotic pathways by preventing accumulation of pro-death Δ*N*-Bcl-xL and enhancing production of pro-survival Bcl-xL. Although limited information exists explaining the direct mechanisms of soy isoflavone-mediated Bcl-xL upregulation, soy isoflavone may interact with estrogen response elements (ERE) controlling Bcl-xL transcription. The *bcl-x* gene, BCL2L1, has been determined to contain an ERE at the 3′ untranslated region, indicating the target of transcriptional regulation via estrogen [138]. As soy isoflavones and 17β-estradiol both bind to estrogen receptor β [131,139], soy isoflavones may enhance Bcl-xL expression through transcriptional regulation by targeting EREs on BCL2L1. 

In addition, Jiang et al. orally delivered various concentrations (20, 40, and 80 mg/kg body weight) of genistein, an abundantly found isoflavone in legumes including soybeans, to rat pups for 12 days and studied the effects of genistein in isoflurane-induced neurodegeneration [137]. Isoflurane inhalation increased fluoro-jade positive degenerating neurons in the hippocampal CA1, CA3, and dentate gyrus regions whereas genistein treatment significantly protected from this neuronal loss in dose dependent manner. Animals supplemented with genistein showed upregulation of Bcl-xL and Bcl-2, and down regulation of Bad and Bax protein levels in the brain leading to improvement in cognitive behaviors [137]. This group further found that genistein treatment significantly increased AKT and phosphorylated AKT protein levels [137]. AKT, protein kinase B, is a serine/threonine kinase that is responsible for the phosphorylation of pro-apoptotic Bad and Bax [140,141,142]. The phosphorylation status of Bad and Bax influences heterodimerization with anti-apoptotic Bcl-2 proteins such as Bcl-xL [141,143]. 

Orally delivered soy isoflavones prevented loss of Bcl-xL protein in the rat brain after intraperitoneal injection of amyloid-β peptides (Aβ) indicating the therapeutic potential of soy isoflavones in the treatment of patients with Alzheimer’s disease [108]. In this study, rats were gavaged with soybean isoflavone (80 mg/kg/day) for 14 days, then Bcl-xL protein levels were quantified using brain tissue. Soybean isoflavone fed group were resistant against Aβ-mediated Bcl-xL loss. Although molecular mechanisms of soy isoflavone-mediated neuroprotection against Aβ-induced oxidative damage are unclear, upregulation of Bcl-xL protein may be beneficial to block activation of Aβ. Conversion of amyloid precursor protein (APP) to active Aβ peptides requires proteolytic cleavage by proteases such as secretases and caspases [144,145,146,147]. Since Bcl-xL binds to the apoptosome and blocks activation of down-stream caspases in the intrinsic apoptotic pathway [148,149], increasing Bcl-xL protein expression by soy isoflavone may further arrest caspase-dependent post-translational modification of APP. 

## 3. Ginseng and Ginsenosides

*Panax ginseng* has been used as a traditional medicine in Asia. Ginseng contains carbohydrates, oils, and amino acids. Most importantly, steroid derivatives of ginseng, ginsenosides (also called ginseng saponin), are reported to be the pharmaceutically functional molecule [150,151]. Antioxidant, anti-inflammatory, and anticancer effects of ginseng have been reported by various laboratories [152,153,154,155]. Moreover, neuroprotective effects of ginseng have been widely documented using both in vivo and in vitro models. Panaxadiol saponins of gisenoside Rb1 have been shown to decrease the loss of interneurons, astrocytes, and microglia in rats treated with kainic acid, an agonist of the AMPA/kainate glutamate receptor, thus preventing kainite-induced experimental seizures [156]. Further research suggests that ginsenoside Rg1 also decreased the MCAO-induced infarct volume in rat brains, indicating therapeutic effects of ginseng to prevent cerebral ischemia [157]. Interestingly, ginseng exerts neuroprotective properties in a concentration dependent manner. A low concentration of panaxatriol saponin treatment attenuated the loss of dopaminergic neurons in zebrafish whereas a high concentration caused neurotoxicity [158]. In vitro approaches using SH-SY5Y cell lines showed similar findings [159]. Ginsenoside Rd significantly increased cell viability at both 1 and 10 µM concentrations against in Parkinson’s Disease models, but it failed to do so at a higher concentration of 50 µM. At both lower concentrations, a decrease in oxidative stress and an increase in superoxide dismutase activity was recorded [159]. Therefore, it is possible that a low or a high concentration of ginsenosides may protect brain cells or facilitate death in malignant cells, respectively. 

Upregulation of Bcl-xL protein by ginseng administration has been reported in various brain-associated disease models including bacterial inflammation, cerebral ischemia, and spinal cord injury [109,110,111,112,113]. Zhang et al. has reported that ginsenoside Rb1 protects the brain against ischemic insult in a Bcl-xL dependent manner [109]. Rats underwent intravenous infusion of ginsenoside Rb1 (6 or 60 µg/µL) for 4 weeks after induction of MCAO. Ginsenoside Rb1 treated animals showed an upregulation of Bcl-xL mRNA and protein in the stroke-affected cortex which was associated with a decrease in cortical infarct size, attenuated apoptosis, and improved navigational ability. This group identified the signal transducer and activator of transcription 5 (STAT5) as being under the control of ginsenoside Rb1-mediated Bcl-xL gene regulation. STAT5 contains a DNA binding domain and acts as a transcription factor; its target genes include Bcl-xL [160]. A Bcl-xL gene with a mutation in the STAT5 response element (STRE) failed to be activated by ginsenoside Rb1, whereas ginsenoside Rb1 treatment increased wild-type Bcl-xL activity by greater than 2 fold. In addition to prevention of cerebral ischemia, Bcl-xL-mediated neuroprotective effects of ginsenoside have been observed in spinal cord injury models [110,111,113] where red ginseng extract administration attenuated the expression of inflammatory cytokines, prevented morphological alteration of the spinal cord and improved rearing and locomotion activity. This group further showed that the neuroprotective properties of red ginseng extract are associated with its ability to upregulate protein levels of Bcl-xL and vascular endothelial growth factor (VEGF) [111,113]. Although the detailed mechanisms were not tested in these studies, VEGF has been reported to be a downstream target of Bcl-xL via the mitogen-activate protein kinase (MAPK) signaling pathway [161]; thus Bcl-xL may promote recovery after CNS injuries by supporting angiogenesis. In addition to transcriptional and translational regulation, ginsenosides may directly bind to Bcl-xL and control its activity. An in silico docking study predicted the interaction between Bcl-xL and ginsenosides, especially with Rg1, Rg2, Rg3, and Rd [162], and Ala142, Glu153, Thr109, Lys16, and Lys20 are reported as the predicted docking sites. In order to show biological significance of ginsenoside docking, verification of the interaction between Bcl-xL and ginsenosides using cells or in tissues will be required in future studies.

## 4. Omega-3 Fatty Acids

Docosahexaenoic acid (DHA) and eicosapentaenoic acid (EPA) are omega-3 (ω-3) fatty acids with 22-carbons and 20-carbons, respectively. Both fatty acids are found in fatty fish such as salmon, trout, and tuna. EPA and DHA are typically available in food or appropriate supplements, but they are also principle metabolites of the essential fatty acid α-linolenic acid. DHA and EPA are structural components of neuronal membranes, thus sufficient intake of omega-3 supports normal brain development [163,164], whereas deficiencies impair neuronal growth and plasticity [165,166,167].

Both in in vitro and in vivo models, application of omega-3 fatty acids has been noted to exert neuroprotection by enhancing the levels of Bcl-xL protein and modifying other Bcl-2 family protein levels [114,115,116,117,118,119,120,168,169]. Fat-1 gene encodes omega-3 fatty acid desaturase, the enzyme responsible for conversion of omega-6 fatty acids to omega-3 fatty acids. Tissues from Fat-1 mice are rich in omega-3 fatty acids such as EPA and DHA [170,171]. Shi et al. reported that cortical neurons isolated from Fat-1 mice were resistant to oxygen glucose deprivation (OGD)-mediated oxidative stress because Bcl-xL levels did not decline [118]. This group also showed that treatment with DHA (10 µM) significantly enhanced Bcl-xL protein in primary neuronal cultures indicating a regulatory effect of both endogenous and exogenous omega-3 fatty acids on Bcl-xL expression [118]. In addition, Fat-1 mice were protected from neuronal loss caused by infusion of amyloid beta (Aβ) 1-42, and primary cortical neurons isolated from Fat-1 embryos retained significantly higher levels of Bcl-xL protein under Aβ challenge [120]. 

Deficiency of thyroid hormone causes neuronal apoptosis associated with downregulation of Bcl-xL protein [116,172,173]. In a model of experimental hypothyroidism in pregnant rats, animals were protected from neuronal apoptosis by prior supplementation with 300 mg of omega-3 fatty acids [116]. Hypothyroid pups born from omega-3 fatty acid fed dams retained cerebellar weight and Bcl-xL protein in the cerebellum [116]. This group further showed that supplementation of omega-3 fatty acid inactivates c-Jun *N*-terminal kinase (JNK) and enhances phosphorylation of AKT and ERK [116]. AKT and ERK may enhance phosphorylation of pro-apoptotic Bcl-2 proteins [140,143] favoring survival by preventing dimerization of Bcl-xL. Interestingly, treatment with neuroprotectin D1, a derivative of omega-3 fatty acids, reverses phosphorylation of Bcl-xL by enhancing protein phosphatase 2A, an enzyme responsible for the dephosphorylation of Bcl-xL [174,175]. Therefore, omega-3 fatty acids may both decrease the functional population of pro-apoptotic Bcl-2 proteins and increase Bcl-xL in the brain favoring neuroprotection.

DHA treatment is reported to protect the brain in in vivo models of Parkinson’s disease [117]. Mice fed with DHA for 2 weeks were protected from dopaminergic cell loss induced by the dopaminergic toxin, 1-methyl-4-phenyl-1,2,3,6-tetra-hydropyridine (MPTP) [117]. Supplementation of DHA significantly increased the mRNA expression of Bcl-xL, leading to the neuroprotective effect. This group also showed downregulation of phosphorylated JNK in the DHA fed group. Although both the JNK pathway and Bcl-xL are known to play a role in apoptosis, it is unclear if DHA independently controls JNK and Bcl-xL expression, or it intervenes with JNK-induced Bcl-xL regulation. Since phosphorylation is an important modification to change the function of Bcl-xL by blocking post-translational cleavage or interaction with pro-apoptotic proteins [103,176,177,178,179], alteration of upstream kinase activities by DHA may regulate apoptosis. 

Moreover, DHA application has been highly effective against oxidative stress-associated damage which is a common process during neurodegeneration [115,119]. Although mechanisms of DHA-mediated epigenetic control were not tested in these reports, DHA-induced augmentation of mRNA expression under oxidative stress is known to occur for the Bcl-xL gene, BCL2L1 in HT22 hippocampal cell line, without changes in Bcl-2 nor Bax gene expression [115]. Thus, DHA-dependent anti-apoptotic effect during oxidative stress may be primarily controlled by the Bcl-xL gene. Finally, treatment with DHA is reported to enhance neurite outgrowth in hippocampal neurons and to prevent loss of neurites during neurotoxicity [120,180]. Since Bcl-xL is required for neurite elongation and branching [21], DHA may be a potent candidate to promote rehabilitation or recovery of the brain after injury.

## 5. Resveratrol

Resveratrol, or 3,4,5-trihydroxystilbene, is part of a class of polyphenolic compounds called stilbenes. Resveratrol is in foods such as cocoa, various berries, and peanuts, but the most prominent sources in the human diet are grapes and grape products, such as wine. Although both red and white wines contain this compound, resveratrol content is markedly higher in red wines [181]. Resveratrol is notable for its role as a potent antioxidant by scavenging free radicals and increasing the activity of endogenous antioxidant enzymes, such as superoxide dismutase [182,183]. Additionally, it has been found to have additional benefits such as cardioprotection, anti-inflammatory, and anti-tumor properties [184,185,186,187]. 

Neuroprotective properties of resveratrol have been reported by various research groups. Administration of resveratrol attenuates brain damage caused by MCAO-induced ischemic stroke in rodents [188,189,190]. Resveratrol also exhibits protective effects in neurodegenerative diseases such as Alzheimer’s and Parkinson’s disease [191,192]. Resveratrol treatment regulated Bcl-xL and NF-κB protein levels in an in vitro Alzheimer’s disease model, and protected rat pheochromocytoma cells, PC12, against Aβ-induced oxidative stress [122]. Moreover, involvement of Bcl-xL in resveratrol-mediated neuroprotection in a cerebral ischemia model was reported [121]. Lanzillotta et al. exposed mouse primary cortical neurons to oxygen-glucose deprivation (OGD) for 3 h, and found that OGD decreased the activity of the Bcl-xL promoter. Interestingly, mutation of the nuclear factor-kappaB (NF-κB) binding site at the Bcl-xL promoter downregulated the basal activity of the Bcl-xL gene indicating NF-κB is an important transcriptional modulator of Bcl-xL gene expression [121]. RelA (also called p65), a subunit of NF-κB, is responsible for inducing Bcl-xL gene expression, but not that of Bcl-2 [193]. Lanzillotta’s group previously reported that acetylation of nuclear factor-kappaB (NF-κB) p50/RelA at Lys310 occurs during ischemia stroke, and deacetylation by a pharmacological inhibitor of acetylase and application of Lys310 mutation attenuates MCAO-induced neuronal injury [194]. Resveratrol activates sirtuin 1, an NAD-dependent deacetylase encoded by SIRT1 gene [195,196], thus resveratrol was applied to reverse acetylation of NF-κB RelA. Consistently, resveratrol enhanced Bcl-xL protein and protected SH-SY5Y cells from prion protein-induced cell death [123]. Seo et al. confirmed that resveratrol attenuates acetylation of p65, which enhances Bcl-xL promoter activity [193]. This group also showed that SH-SY5Y cells transduced with SIRT1 siRNA abolished resveratrol-mediated protection indicating that SIRT1 is the primary target of resveratrol in this model. These studies report that manipulating acetylation state using resveratrol supports recruitment of transcription factors at the Bcl-xL promoter enhancing the ratio of anti-apoptotic to pro-apoptotic gene products; this protects the brain against various apoptotic insults.

Although there are limited reports indicating a relationship between Bcl-xL and resveratrol in CNS disease models, numerous studies have reported that Bcl-2 and other members of Bcl-2 proteins are under the control of resveratrol [189,197,198,199,200,201]. Since apoptotic pathways are orchestrated by a series of highly coordinated actions of Bcl-xL and other Bcl-2 family proteins, it may be critical to dissect functions of Bcl-xL during resveratrol-mediated neuroprotection in future research. 

## 6. Alcohol

Alcohol, specifically ethanol, is a substance known to have varying effects on the brain. Although alcohol sensitivities may vary by dosage, time, cell type, and individual genetic differences [202], it is well-reported that aggressive alcohol usage leads to neurological damage, which is causative of behavioral and cognitive changes [203,204]. In contrast to its neurotoxic effects, moderate consumption of alcohol can be neuroprotective, lowering the risk of ischemic stroke and enhancing cognitive function in human subjects [205,206,207,208]. Similarly, treatment with ethanol during or after MCAO procedure improved energy metabolism and protected rodent brain from ischemic injury [209,210].

Despite limited information regarding the underlying mechanisms of alcohol-mediated neuroprotection, Bcl-2 proteins including Bcl-xL have been reported as important molecular targets [124,125,211,212]. Fu et al. showed upregulation of Bcl-xL protein in ethanol treated animals in an ischemic stroke model [124]. Rats undergoing MCAO-induced transient ischemia followed by reperfusion, had significantly decreased pro-apoptotic protein levels such as Bax, caspase 3, and apoptosis inducing factor (AIF) after administration of 1.5 g/kg ethanol, and the ethanol treatment enhanced anti-apoptotic Bcl-xL and Bcl-2 protein levels. Bcl-xL directly binds to Bax to inhibit Bax-mediated membrane permeabilization [213] and interaction between Bcl-xL and Bax prevents AIF release. Sequestration of pro-apoptotic Bax by Bcl-xL also prevents formation of the apoptosome blocking activation of caspase 3. Since caspase 3 is a key contributor in the conversion of anti-apoptotic Bcl-xL to pro-apoptotic fragmented ∆*N*-Bcl-xL during cerebral stroke [18,65,68], the ability of ethanol to prevent caspase 3 induction and to increase Bcl-xL protein levels directly prevents activation of apoptotic pathways. The same research team has reported that intraperitoneal injection of ethanol (1 g/kg) with normobaric oxygen therapy significantly increased Bcl-xL protein levels and attenuated stroke-induced neurological deficits [125]. However, it is well studied that alcohol consumption can also lead to apoptotic death via decreases in levels of Bcl-2 proteins in the brain [214,215,216]. Ethanol-induced Bcl-xL mRNA expression in brain cells is highly dependent on its concentration [124] and on the length of time of miRNA expression [217], thus determination of the therapeutic window of protection after ethanol treatment will be needed in future projects.

## 7. Conclusions

In this review, we have discussed food sources, nutrients, and phytochemicals that exert neuroprotection by regulating Bcl-xL (Table 2). Unlike surgical procedures or pharmacological approaches, nutritional therapy may not directly cure CNS disease or immediately relieve symptoms. However, nutritional intervention may delay propagation of neurotoxic signaling in the brain, facilitate recovery of damaged neurons, or support medical treatment by controlling an important molecular target that is essential for mitochondrial energy production, synaptic transmission, neurite outgrowth, and neuronal survival. Here, we suggest that nutrients are participants in molecular events and pathways, and are capable of regulating the protein target Bcl-xL and directly or indirectly other Bcl-2 family members via controlling transcription, translation, post-translational modification, and direct binding. Nutrients with regulatory potential may act synergistically with existing medications to reduce the risk of CNS disease.

## Figures and Tables

**Table 1:** Neuroprotective properties of Bcl-xL in CNS-associated disease models.

CNS-Associated Disorders	References
Ischemic stroke	[18,30,31,32,33,34,35]
Spinal cord injury	[36,37,38,39,40]
Alzheimer	[41,42,43,44]
Parkinson	[45,46,47,48,49,50]
Anxiety and Depression	[51,52,53]
Traumatic Brain injury	[54,55,56]
Spinal Muscular Dystrophy	[57,58,59,60,61]
Schizophrenia	[62,63,64]

**Table 2:** A list of dietary compounds that regulate Bcl-xL in the brain.

Nutrients and Foods	Roles	References
High soy diet	Upregulation of Bcl-xL mRNA and protein in rats	[106]
High soy diet	Upregulation of Bcl-xL mRNA and protein in rats MCAO model	[107]
Orally gavaged soy isoflavones	Upregulation of Bcl-xL protein against Aβ-induced neurotoxicity in rats	[108]
Intravenous infusion of ginsenoside	STAT5-dependent upregulation of Bcl-xL protein in rodent ischemia models (rats and gerbils) and primary cortical neurons	[109]
Intravenous infusion of dihydroginsenoside Rb1	Upregulation of Bcl-xL mRNA in spinal cord injury and cerebral ischemia models	[110]
Oral administration of red ginseng extract	Upregulation of Bcl-xL protein in rat spinal cord injury model	[111]
Oral administration and intravenous infusion of ginsenoside	Upregulation of Bcl-xL protein in mice during pneumococcal infection	[112]
Intravenous infusion of ginsenoside	Upregulation of Bcl-xL protein in rat spinal cord injury model	[113]
DHA or neuroprotectin D1 treatment in vitro	Upregulation of Bcl-xL gene in HN cell line	[114]
DHA treatment in vitro	Upregulation of Bcl-xL mRNA in HT22 cell line against MNNG-induced toxicity	[115]
Oral supplementation of DHA and EPA	Upregulation of Bcl-xL protein in hypothyroid rats	[116]
Oral supplementation of DHA	Upregulation of Bcl-xL mRNA in mice brains after challenge with dopaminergic toxin	[117]
Application with Fat-1 mouse model	Cortical neurons isolated from Fat-1 mice, genetically modified model with high endogenous omega-3 fatty acid levels, retained Bcl-xL protein against OGD	[118]
DHA treatment in vitro	Upregulation of Bcl-xL protein in primary cortical neurons against oxyhemoglobin treatment	[119]
Primary cortical neurons isolated from Fat-1 mice	Upregulation of Bcl-xL protein in primary cortical neurons against Aβ 1-42 treatment	[120]
Intraperitoneal delivery of resveratrol	Upregulation of acetylation of H3 histones on Bcl-xL promoter in mice	[121]
Resveratrol treatment in vitro	Upregulation of Bcl-xL protein in PC12 cells	[122]
Resveratrol treatment in vitro	Upregulation of Bcl-xL protein in SH-SY5Y cells	[123]
Intraperitoneal injection of ethanol	Upregulation of Bcl-xL protein in rat MCAO model	[124]
Intraperitoneal injection of ethanol	Upregulation of Bcl-xL protein in rat MCAO model	[125]
α-Tocopherol treatment in vitro	Downregulation of Bax/Bcl-xL protein ratio in PC 12 cells	[126]
Methyl donor deficient diet (no folate or vitamin B_12_)	Downregulation of Bcl-xL protein via miR-124-mediated Stat3 inhibition	[127]
